# Dermatofibrosarcoma Protuberans of Scalp With Cervical Lymph Node Metastasis

**DOI:** 10.1080/13577140410001679257

**Published:** 2004-03

**Authors:** Pawan Lal, Arun Goel, A. K. Mandal

**Affiliations:** 1 Department of Surgery Maulana Azad Medical College Associated Lok Nayak G.B. Pant Hospitals New Delhi 110002 India; 2 Department of Plastic Surgery Maulana Azad Medical College Associated Lok Nayak G.B. Pant Hospitals New Delhi 110002 India; 3 Department of Pathology Maulana Azad Medical College Associated Lok Nayak G.B. Pant Hospitals New Delhi 110002 India; 4 C–63 Preet Vihar Delhi 110092 India

## Abstract

Dermatofibrosarcoma protuberans (DFSP) is an uncommon, slow growing and locally aggressive tumor of the skin with a high rate of recurrence even after supposedly wide excision. The reports of regional lymph node metastasis and distant metastasis are very rare. Because of the extreme rarity of these cases with metastasis, the experience with management of such patients is very limited. A case of recurrent DFSP of scalp, with metastasis to the regional lymph nodes, in a 17-year-old boy is reported here. This is the second case of DFSP involving scalp and 16th case of DFSP of all sites metastasizing to the regional lymph nodes reported in literature. The patient was treated with wide excision of the lesion and ipsilateral radical neck dissection (including excision of overlying involved skin).


Sarcoma, March 2004, VOL. 8, NO. 1, 43–45

Taylor & Francis healthsciences

CASE REPORT

### Dermatofibrosarcoma protuberans of scalp with cervical lymph node metastasis

PAWAN LAL[1], ARUN GOEL[2] & A.K. MANDAL[3]

*Departments of [1]Surgery, [2]Plastic Surgery and [3]Pathology, Maulana Azad Medical College and Associated Lok Nayak and G.B. Pant Hospitals, New Delhi 110002, India*

**Abstract**
Dermatofibrosarcoma protuberans (DFSP) is an uncommon, slow growing and locally aggressive tumor of the skin with a high rate of recurrence even after supposedly wide excision. The reports of regional lymph node metastasis and distant metastasis are very rare. Because of the extreme rarity of these cases with metastasis, the experience with management of such patients is very limited. A case of recurrent DFSP of scalp, with metastasis to the regional lymph nodes, in a 17-year-old boy is reported here. This is the second case of DFSP involving scalp and 16th case of DFSP of all sites metastasizing to the regional lymph nodes reported in literature. The patient was treated with wide excision of the lesion and ipsilateral radical neck dissection (including excision of overlying involved skin).

**Key words:** *dermatofibrosarcoma protuberans, lymph nodal, metastasis*

### Case report

A 17-year-old boy was admitted to Lok Nayak Hospital with the chief complaints of a gradually increasing swelling in the right occipito-temporal region of the scalp for 11 years and multiple swellings in the ipsilateral neck for 1 year. His parents had first noticed a small $2 \times 2$-cm swelling on the right side of the scalp when the patient was 6 years old. This swelling was excised at a small peripheral medical center when the patient was 10 years of age. Till then, it increased in size very slowly. The parents did not have any medical record or histopathological report. The swelling reappeared about 1 year after the excision. Since then, it had been again increasing in size slowly and painlessly. One year prior to presentation to us, the patient also noticed a few swellings in the right half of the neck. These were also painless and slow to increase in size.

General physical and systemic examination of the patient showed no abnormality or unusual feature. All the findings were localized to head and neck. Examination of the scalp showed an $8 \times 6 \times 4$-cm fungating, non-tender swelling over the right temporo-parieto-occipital region. It was mobile over the scalp and firm in consistency. There was another swelling, $4 \times 5$ cm in size, on the right side of the neck. It was fixed to sternocleidomastoid muscle and adherent to overlying skin. There was no ulceration in the neck (Fig. 1). Multiple lymph nodes were palpable in the posterior triangle of the neck. They were mobile, firm in consistency, non-tender with the largest about 2 cm in size. A clinical diagnosis of soft tissue tumor of the scalp with metastasis in the neck was made. Skiagram of the chest showed no evidence of metastasis. CT Scan of head and neck was suggestive of a malignant soft tissue tumor in the scalp with metastasis in the neck with normal calvarium and intracranial structures. Fine needle aspiration cytology from the neck tumor was reported to be a malignant mesenchymal lesion suggestive of DFSP.

The patient underwent wide excision of the primary on the scalp with a 4-cm margin all around. The neck nodes were managed by radical neck dissection on the right side of the neck with wide excision (taking a 4-cm margin all around) of the involved adherent skin. The scalp lesion was adherent to the periosteum over a 2.5-cm diameter area in the central region of the tumor, and hence the periosteum also required removal. The rest of the area had a healthy and intact periosteum. The denuded bone was healthy. The whole of the defect on the scalp and the neck was covered with a split

Correspondence to: Dr. Pawan Lal, C–63, Preet Vihar, Delhi 110092, India. Tel.: +91-11-2014727; E-mail: pawanlal@vsnl.com

1357-714X print/1369–1643 © 2004 Taylor & Francis Ltd
DOI: 10.1080/13577140410001679257

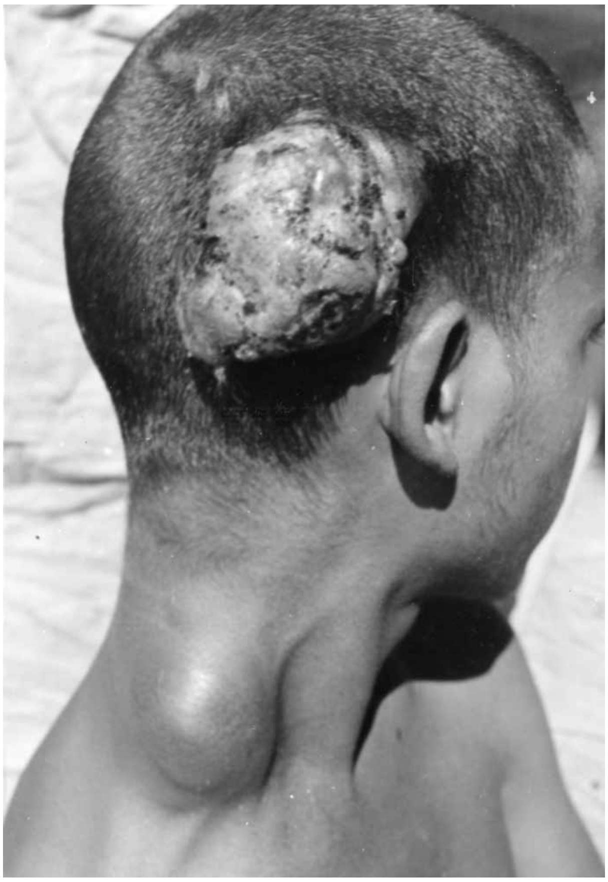

**Fig. 1.** *Pre-operative photograph showing the primary scalp tumor with cervical lymph node metastases.*

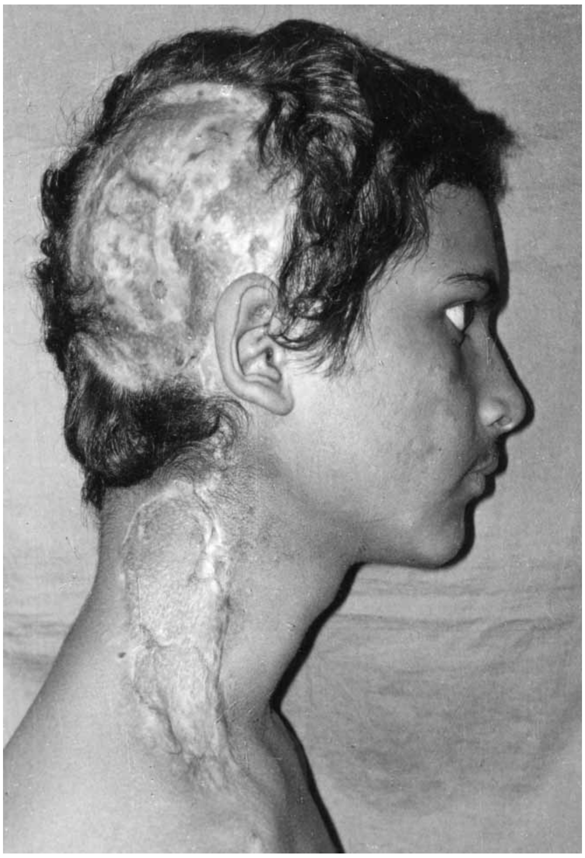

**Fig. 2.** *Five weeks postoperative photograph showing complete healing of the scalp and neck areas.*

skin graft from the thigh. As expected, there was a small graft loss over the denuded bone, but conservative management using dressings with antibiotic ointment and petrolatum gauze on every third day allowed the area to develop granulation tissue and spread of the graft. Complete healing occurred in 5 weeks (Fig. 2). The patient is disease-free in the follow-up period of 18 months duration.

Histopathological examination of the excised scalp specimen showed the tumor cells to be spindle-shaped and at places with a storiform pattern (Fig. 3). The tumor was poorly circumscribed and showed infiltrative margins. At a few places, the storiform pattern gave way to more fascicular areas. The overlying skin showed ulceration and infiltration by the tumor cells. The neck specimen showed histopathological features similar to the scalp lesion. Although, there was no ulceration, the tumor cells were reaching up to the epidermis. The lymph nodes from the posterior triangle of neck showed tumor metastasis (Fig. 4).

### Discussion

Dermatofibrosarcoma protuberans (DFSP) is a tumor with high rate of local recurrence and an extremely low but definite risk of metastasis. Hematogenous spread is very rare and lymphatic

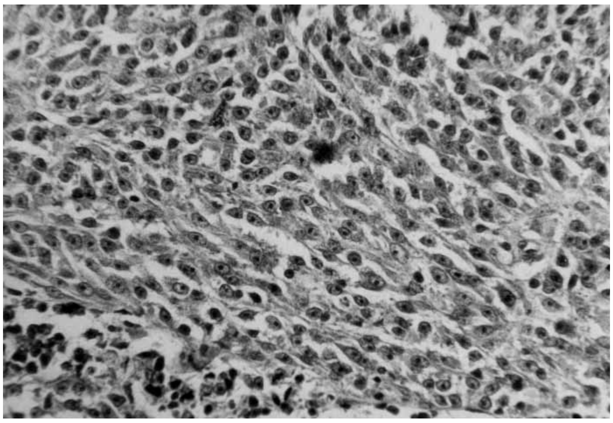

**Fig. 3.** *High power detail (×40) of scalp specimen with spindle-shaped cells in storiform pattern (H&E stain).*

involvement is even rarer. In an extensive review of literature by Rutgers *et al.*[1] involving 913 cases, 11 were found to have regional lymph node metastasis. They even tabulated the various parameters of all these cases reported by various authors. However, a later article by Mavili *et al.*[2] reported their own case to be the tenth. Study of these two reports, their similarities and discrepancies, and other case reports published prior to or later than these review articles brings the figure to 15, with the present author's case being the 16th case with lymph node metastasis in a case of DFSP. The articles reporting such cases

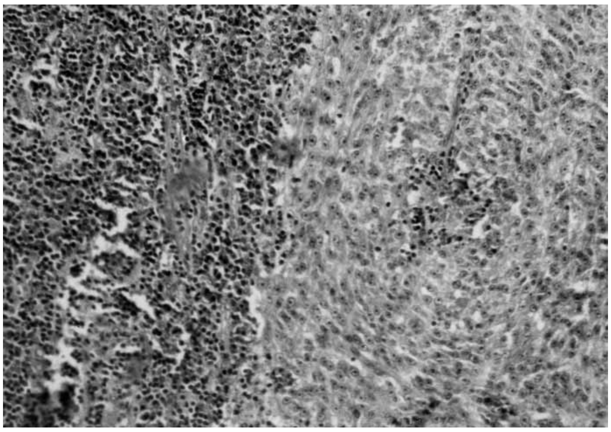

**Fig. 4.** *Cervical lymph node showing tumor cells (×10) (H&E stain).*

according to their year of publication are those by Gentele in 1951,[3] Woolridge in 1957,[4] Waldermann *et al.* in 1958,[5] Przybore *et al.* in 1959,[6] Fisher *et al.* in 1966,[7] Brenner *et al.* in 1975,[8] Kahn *et al.* in 1978,[9] Hausner *et al.* in 1978,[10] Volpe *et al.* in 1983,[11] Petoin *et al.* in 1985,[12] Hirabayashi *et al.* in 1989,[13] Mavili *et al.* in 1994[2] and Lal *et al.* in 1999.[14] Of these articles, only the case reported by Hirabayashi *et al.*[13] had involvement of the scalp as the primary site. Thus, the case reported by the present authors becomes the second reported case of DFSP of the scalp with metastasis to the regional cervical lymph nodes.

In view of the extreme rarity of regional metastasis, prophylactic lymph node dissection is not advocated, while it is but natural to go ahead with it if there is a positive involvement. In the present case, since the overlying skin in the neck was also adherent over a large area, a wide excision of the skin was also done and the resultant defect covered with split skin graft.

### Acknowledgement

Thanks to Mr. Subhankar Ghosh, Photographer, Department of Pathology, Maulana Azad Medical College, New Delhi, India.

### References

1. Rutgers EJTh, Kroon Bin BR, Albus-Lutter CE, Gortzak E. Dermatofibrosarcoma protuberans treatment and prognosis. *Eur J Surg Oncol* 1992; 18: 241–8.
2. Mavili ME, Gursu KG, Gokoz A. Dermatofibrosarcoma with lymph node involvement. *Ann Plast Surg* 1994; 32(4): 438–40.
3. Gentele H. Malignant, fibroblastic tumors of the skin. *Acta Dermatol Venereol* 1951; 31: 91–132.
4. Woolridge WE. Dermatofibrosarcoma protuberans: a tumor too lightly considered. *Arch Dermatol* 1957; 75: 132–5.
5. Waldermann F, Hagedoorn M. Klinik and Pathologic des dermatofibrosarcoma protuberans. *Z Hautkr* 1958; 60: 1886–94.
6. Przybora LA, Wojnerowicz C. Malignancy of dermatofibrosarcoma protuberans and report of 2 cases with lymph gland metastases. *Oncologia* 1959; 12: 236–54.
7. Fisher ER, Hellstrom HR. Dermatofibrosarcoma with metastasis simulating Hodgkin's disease and reticulum cell sarcoma. *Cancer* 1966; 19: 1165–71.
8. Brenner W, Schaefler K, Chhabra H, Postel A. Dermatofibrosarcoma protuberans metastatic to a regional lymph node. Report of a case and review. *Cancer* 1975; 36: 1897–902.
9. Kahn LB, Saxe N, Gordon W. Dermatofibrosarcoma with lymph node and pulmonary metastasis. *Arch Dermatol* 1978; 114(4): 599–601.
10. Hausner RJ, Vargas-Cortes F, Alexander RW. Dermatofibrosarcoma protuberans with lymph node involvement: a case report of simultaneous occurrence with an atypical fibroxanthoma of skin. *Arch Dermatol* 1978; 114(1): 88–91.
11. Volpe R, Carbone A. Dermatofibrosarcoma protuberans metastatic to lymph nodes and showing a dominant histocytic component. *Am J Dermatopathol* 1983; 5(4): 327–34.
12. Petoin DS, Verola O, Banzet P, Dufourmantel Cl, Servant JM. Dermatofibrosarcoma de Darier et Ferrand. Etude de 96 cas sur 15 ans. *Chirurgie* 1985; 111: 132–8.
13. Hirabayashi S, Kajikawa A, Kanazawa K, Mimoto K. Dermatofibrosarcoma protuberans with regional lymph node metatstasis: a case report. *Head Neck* 1989; 11(6): 562–4.
14. Lal P, Sharma R, Mohan H, Sekhon MS. Dermatofibrosarcoma protuberans metastasizing to lymph nodes: a case report and review of literature. *J Surg Oncol* 1999; 72: 178–80.
